# Supplementary material for: Electrocorticography and stereo EEG provide distinct measures of brain connectivity: implications for network models
Source: Brain Commun. 2021 Jul 11;3(3):fcab156. doi: 10.1093/braincomms/fcab156 (PMC8361393; doi:10.1093/braincomms/fcab156)
Supplement: fcab156_Supplementary_Data [file fcab156_supplementary_data.docx]

**Supplementary materials for:**

Electrocorticography and stereo EEG provide distinct measures of brain connectivity: Implications for network models

John M. Bernabei^1,2^, T. Campbell Arnold^1,2^, Preya Shah^1,2^, Andrew Revell^1,2^, Ian Z. Ong^1,2^, Lohith G. Kini^1,2^, Joel M. Stein^3^, Russell T. Shinohara^4,5,6^, Timothy H. Lucas^7^, Kathryn A. Davis^2,8^, Danielle S. Bassett^1,7,8,9,10,11,12^, Brian Litt^1,2,7,8^

**Affiliations:**

^1^Department of Bioengineering, University of Pennsylvania, Philadelphia, PA, 19104

^2^Center for Neuroengineering & Therapeutics, University of Pennsylvania, Philadelphia PA 19104

^3^Department of Radiology, Hospital of the University of Pennsylvania, Philadelphia, PA, 19104

^4^Department of Biostatistics, Epidemiology, & Informatics, University of Pennsylvania, Philadelphia, PA, 19104

^5^Statistics in Imaging and Visualization Center, University of Pennsylvania, Philadelphia, PA, 19104

^6^Center for Clinical Epidemiology and Biostatistics, University of Pennsylvania, Philadelphia, PA, 19104

^7^Department of Neurosurgery, Hospital of the University of Pennsylvania, Philadelphia, PA, 19104

^8^Department of Neurology, Penn Epilepsy Center, Hospital of the University of Pennsylvania, Philadelphia, PA, 19104 USA

^9^Department of Electrical & Systems Engineering, University of Pennsylvania, Philadelphia, PA, 19104

^10^Department of Physics & Astronomy, University of Pennsylvania, Philadelphia, PA, 19104

^11^Department of Psychiatry, University of Pennsylvania, Philadelphia, PA, 19104

^12^The Santa Fe Institute, Santa Fe, NM, 87501

To whom correspondence should be addressed: [John.Bernabei@pennmedicine.upenn.edu](mailto:John.Bernabei@pennmedicine.upenn.edu)

Contents:

Table S1: Extended patient information

Figure S1: Anatomic distribution of electrodes is similar in ECoG and SEEG

Figure S2: Network connectivity in alternate frequency bands

Figure S3: Distinguishability of resected versus spared tissue in alternate frequency bands

| Portal ID | Surg. | Type. | Lat. | Les. | Age | Sex | Total  Node | Depth  Node | Total Res. | Depth  Res. |
| --- | --- | --- | --- | --- | --- | --- | --- | --- | --- | --- |
| HUP65_phaseII | RES | ECoG | R | LES | 36 | M | 64 | 0 | 15 | 0 |
| HUP68_phaseII | RES | ECoG | R | NL | 28 | F | 83 | 0 | 24 | 0 |
| HUP74_phaseII | RES | ECoG | L | LES | 25 | F | 108 | 17 | 53 | 17 |
| HUP78_phaseII | RES | ECoG | L | LES | 54 | M | 97 | 0 | 20 | 0 |
| HUP82_phaseII | RES | ECoG | R | LES | 56 | F | 83 | 5 | 40 | 3 |
| HUP88_phaseII | RES | ECoG | L | LES | 35 | F | 53 | 7 | 7 | 0 |
| HUP89_phaseII | RES | ECoG | R | LES | 29 | M | 95 | 7 | 9 | 5 |
| HUP094_phaseII | RES | ECoG | R | NL | 48 | F | 81 | 14 | 2 | 0 |
| HUP097_phaseII | RES | ECoG | L | NL | 39 | F | 91 | 7 | 15 | 1 |
| HUP099_phaseII_D01 | RES | ECoG | R | LES | 20 | F | 105 | 12 | 28 | 6 |
| HUP105_phaseII | RES | ECoG | R | LES | 39 | M | 54 | 7 | 4 | 0 |
| HUP106_phaseII | RES | ECoG | L | NL | 45 | F | 114 | 14 | 9 | 6 |
| HUP107_phaseII | RES | ECoG | R | NL | 36 | M | 114 | 17 | 22 | 8 |
| HUP111_phaseII_D02 | RES | ECoG | R | NL | 40 | F | 100 | 11 | 6 | 3 |
| HUP116_phaseII | ABL | SEEG | R | LES | 59 | F | 34 | 34 | 5 | 5 |
| HUP117_phaseII | RES | SEEG | L | LES | 39 | M | 29 | 29 | 3 | 3 |
| HUP125_phaseII_D04 | ABL | ECoG | L | NL | 57 | M | 108 | 20 | 8 | 8 |
| HUP126_phaseII_D01 | ABL | ECoG | L | NL | 26 | F | 123 | 37 | 9 | 9 |
| HUP138_phaseII | ABL | SEEG | L | LES | 38 | M | 71 | 71 | 3 | 3 |
| HUP140_phaseII_D02 | ABL | SEEG | L | NL | 47 | F | 53 | 53 | 5 | 5 |
| HUP144_phaseII | RES | SEEG | R | LES | 31 | M | 86 | 86 | 15 | 15 |
| HUP146_phaseII | RES | SEEG | R | NL | 16 | M | 86 | 86 | 7 | 7 |
| HUP148_phaseII_D02 | ABL | SEEG | L | LES | 23 | M | 69 | 69 | 7 | 7 |
| HUP157_phaseII | ABL | SEEG | L | NL | 25 | M | 116 | 116 | 6 | 6 |
| HUP160_phaseII | RES | SEEG | R | NL | 45 | F | 65 | 65 | 11 | 11 |
| HUP164_phaseII | ABL | SEEG | L | LES | 34 | F | 131 | 131 | 3 | 3 |
| HUP165_phaseII | ABL | SEEG | R | NL | 21 | F | 151 | 151 | 10 | 10 |
| HUP173_phaseII | RES | SEEG | R | LES | 24 | F | 87 | 87 | 17 | 17 |
| HUP177_phaseII | RES | SEEG | R | NL | 42 | F | 139 | 139 | 16 | 16 |
| HUP181_phaseII_D02 | ABL | SEEG | L | LES | 31 | F | 103 | 103 | 6 | 6 |
| HUP185_phaseII | ABL | SEEG | L | LES | 38 | M | 93 | 93 | 9 | 9 |
| HUP187_phaseII | ABL | SEEG | R | NL | 25 | M | 77 | 77 | 7 | 7 |
| HUP190_phaseII | RES | SEEG | L | NL | 25 | M | 117 | 117 | 25 | 25 |

**Table S1: Extended patient information.** Column 1) ieeg.org portal ID where full-length records are freely available & searchable. Column 2) Surgery type – RES: resection, ABL: laser ablation. Column 3) Laterality (Right/Left). Column 4) Pre-op lesion status – LES: lesional, NL: non—lesional. Column 5) Sex (Male/Female). Column 6) Total number of nodes in grey matter (GM). Column 7) Total number of GM nodes sampled by depth electrodes. Column 8) Total number of resected GM nodes. Column 9) Total number of resected GM nodes sampled by depth electrodes.


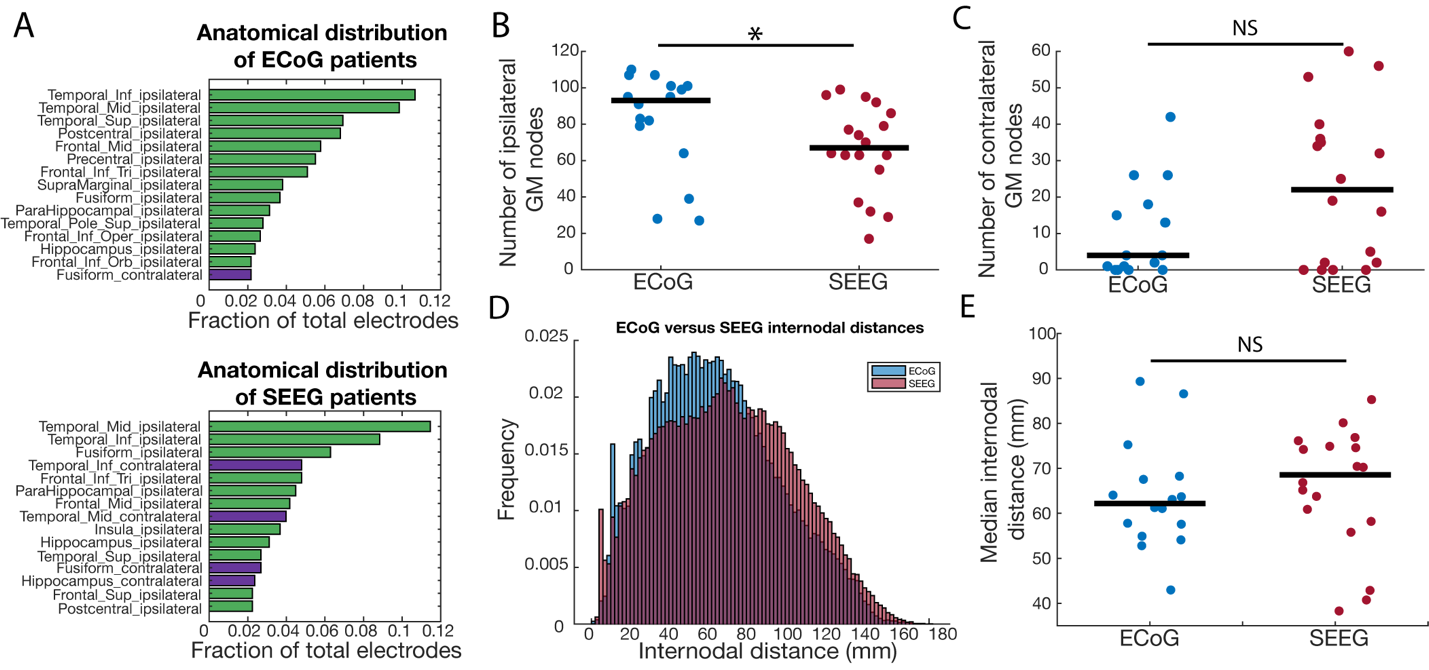


**Figure S1. Anatomic distribution of electrodes is similar in ECoG and SEEG:** (A) The top 15 ranked AAL regions in terms of total number of electrodes across patients. Green: ipsilateral, Purple: contralateral. Abbreviations: Mid: middle, Inf: inferior, Sup: superior, Tri: pars triangularis, Oper: pars opercularis, Orb: pars orbitalis. (B) The number of grey matter nodes ipsilateral to the resection zone was higher in ECoG (median: 93) versus SEEG (median: 64), (rank-sum test, p = 0.022). (C) Comparing the number of grey matter nodes contralateral to the resection zone between ECoG (median: 4), and SEEG (median: 25) did not reach statistical significance (rank-sum test, p = 0.057). (D) Distribution histogram of internodal distances in ECoG (blue) and SEEG (red). (E) The median internodal distance in each patient was not significantly different between ECoG (median: 62.2) versus SEEG (median: 70.2), (rank-sum test, p = 0.24).


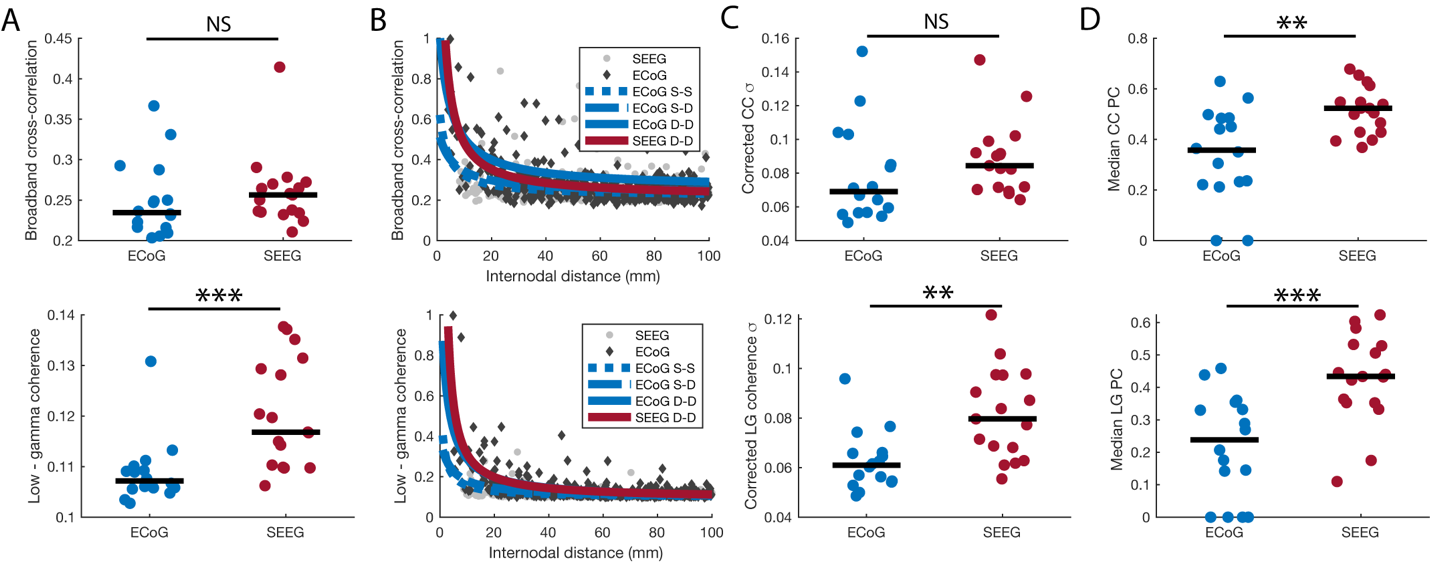


**Figure S2. Network connectivity in alternate frequency bands:** Data for broadband cross-correlation (CC) are on the top row, and low-gamma (LG) coherence is on the bottom row. (A) Median connectivity values were not significantly different between ECoG and SEEG for broadband cross-correlation (rank-sum test p = 0.17), but SEEG had higher low-gamma coherence (rank-sum test p = 1.9e-4). (B) We fit a nonlinear regression model to ECoG surface – surface (dotted blue line), surface – depth (dashed blue line), and depth – depth connections (solid blue line), as well as SEEG depth – depth connections (solid red line). (C) After correcting for internodal distance, the standard deviation of edge weights remained higher in SEEG versus ECoG for low-gamma coherence (LG rank-sum test p = 0.0013) but not broadband cross-correlation (CC rank-sum test p = 0.10). (D) After correcting for internodal distance, the median participation coefficient remained higher in SEEG versus ECoG (CC rank-sum test p = 0.0026, LG rank-sum test p = 8.5e-4). Abbreviations: NS = not significant, S-S: surface – surface, S-D: surface – depth, D-D: depth – depth, SD: standard deviation, ** = p < 0.01, *** = p < 0.001.

**
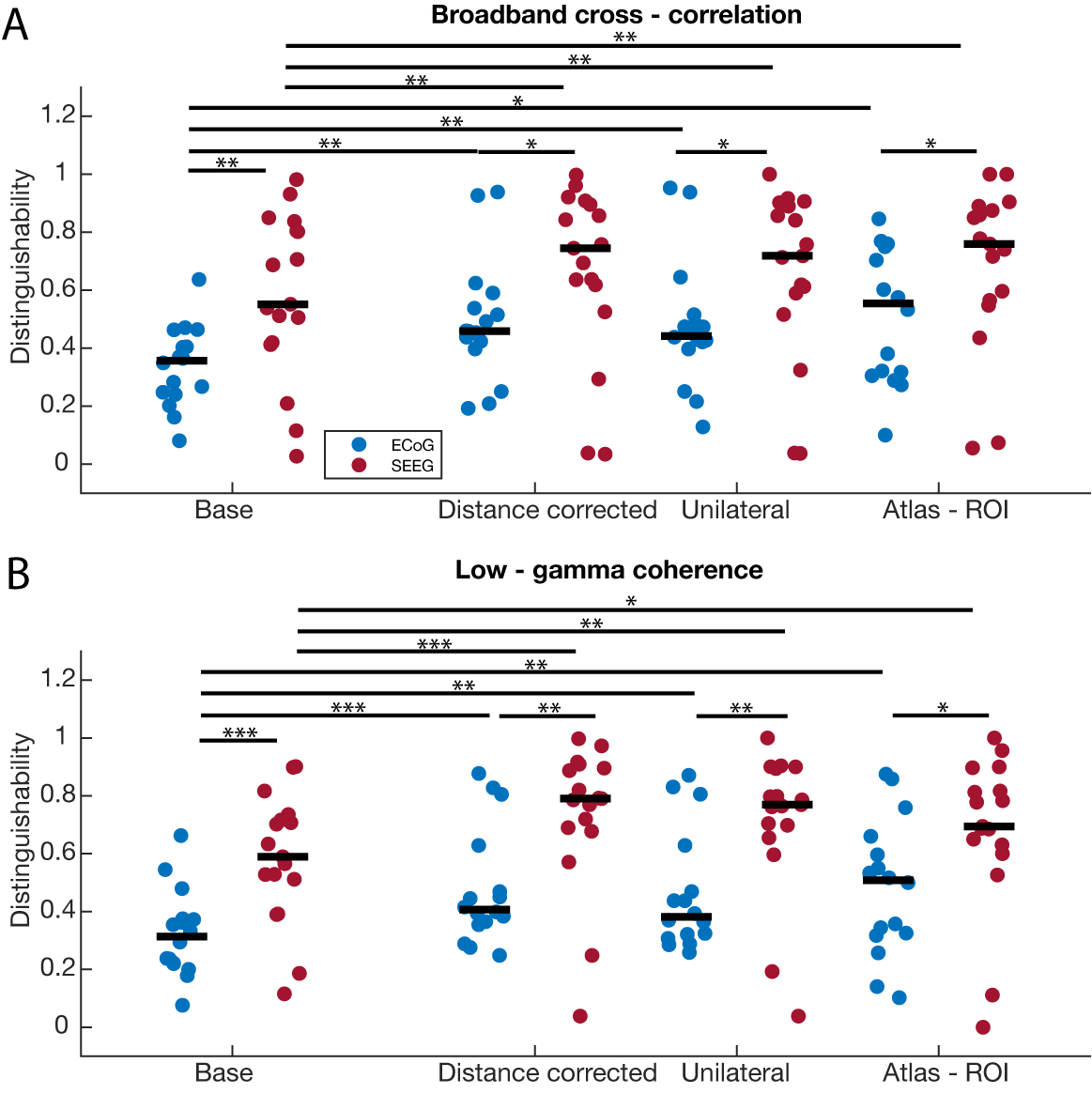
**

**Figure S3. Distinguishability of resected versus spared tissue in alternate frequency bands:** We computed distinguishability for the different networks derived from ECoG and SEEG (Figure 4A), including only grey matter (GM), distance corrected (DC), networks with contralateral nodes eliminated (UL), and networks with atlas-level ROI (AR). (A) For broadband cross-correlation in each condition, SEEG networks had a higher D_rs_ value than ECoG. Each condition in ECoG and SEEG also had a higher D_rs_ value than not accounting for internodal distance. GM ECoG vs GM SEEG: (rank-sum test p = 0.0042), DC ECoG vs DC SEEG: (rank-sum test p = 0.024), UL ECoG vs UL SEEG: (rank-sum test p = 0.029), AR ECoG vs AR SEEG: (rank-sum test p = 0.029), DR/UL/MR ECoG vs GM ECoG: (sign-rank test p = 0.0023/0.0072/0.0131), DC/UL/AR SEEG vs GM SEEG: (sign-rank test p = 0.0012/0.0019/0.0075). (B) For low – gamma coherence: SEEG networks had a higher D_rs_ value than ECoG. Each condition in ECoG and SEEG also had a higher D_rs_ value than not accounting for internodal distance. GM ECoG vs GM SEEG: (rank-sum test p = 9.8e-4), DC ECoG vs DC SEEG: (rank-sum test p = 0.0059), UL ECoG vs UL SEEG: (rank-sum test p = 0.0090), AR ECoG vs AR SEEG: (rank-sum test p = 0.014). DC/UL/AR ECoG vs GM ECoG: (sign-rank test p = 0.0009/0.0027/0.0052), DC/UL/AR SEEG vs GM SEEG: (sign-rank test p = 0.0007/0.0010/0.0245).
